# Inhibition of *Streptococcus mutans* biofilm formation and virulence by natural extract Stevioside

**DOI:** 10.3389/fmicb.2025.1675322

**Published:** 2025-10-02

**Authors:** Rong Ma, Peiling Wang, Yuanyuan Zhang, Yimin Wang, Jinpu Chu

**Affiliations:** Department of Stomatology, The First Affiliated Hospital of Zhengzhou University, Zhengzhou, China

**Keywords:** stevioside, *Streptococcus mutans*, biofilm, virulence factor, gene expression

## Abstract

**Objective:**

This study aimed to investigate the effect of the natural extract of Stevioside on biofilm formation and cariogenic virulence factors of *Streptococcus mutans (S. mutans)*, and to explore its mechanism of action preliminarily, with the goal of identifying a safer and more effective non-cariogenic sweetener.

**Methods:**

The inhibitory effect of Stevioside on the growth of *S. mutans* biofilm was detected by crystal violet staining. The acid production capacity of *S. mutans* biofilms was evaluated by measuring the pH values and lactic acid contents. The bacterial viability within the biofilms was determined using the plate counting method. Scanning electron microscopy (SEM) and laser confocal microscopy (CLSM) were used to observe the biofilm structure, and the fluorescence intensity of live and dead bacteria and biofilm thickness were further analyzed. The anthrone sulfuric acid method quantified the production of Soluble Extracellular Polysaccharide (SEPS) and Insoluble Extracellular Polysaccharide (IEPS) in biofilm. Furthermore, real-time fluorescence quantitative PCR (RT-qPCR) was used to detect the expression of genes related to biofilm growth, acid production, acid tolerance, exopolysaccharide synthesis, two-component signal and quorum sensing.

**Results:**

Stevioside significantly inhibited the formation of *S. mutans* biofilm, and reduced acid production, bacterial activity and EPS production. SEM and CLSM confirmed the reduction of the dense three-dimensional structure of biofilm. In addition, compared with sucrose, the expression of related genes was down-regulated in Stevioside.

**Conclusion:**

Stevioside inhibits *S. mutans* virulence factors and biofilms and is a promising natural sucrose substitute for preventing dental caries.

## 1 Introduction

Dental caries is a biofilm-mediated disease characterized by the progressive destruction of dental hard tissue. The development and progression of this multifactorial condition are regulated by diet and various non-infectious factors (Pitts et al., 2021). The existence of microorganisms, particularly bacteria, is essential for the development of dental caries, with *S. mutans* is widely recognized as the primary cariogenic bacterium. *S. mutans* possesses the capability to adhere and form biofilms, synthesize extracellular polysaccharides and intracellular polysaccharides, and produce acid and acid resistance (Fujita et al., 2011). Modern etiology suggests compared with the planktonic state, biofilm has significant advantages in growth mode, metabolic activity, functional expression (Bowen et al., 2018). As a three-dimensional natural barrier, biofilm can promote the interaction within microorganisms, and protect microorganisms from the external environment (Lemos et al., 2019). Additionally, food is a key factor affecting biofilm formation. Sucrose is the most cariogenic carbohydrate and provides metabolic substrates for *S. mutans* (Klein et al., 2009).

First, *S. mutans* completes the initial surface colonization through its cell wall surface protein SpaP and multiple molecules on the tooth surface (such as salivary lectin glycoprotein). This process is called sucrose-independent adhesion (Yang et al., 2019). However, sucrose-dependent adhesion plays a more critical role in the formation and maturation of biofilms (Tao and Tanzer, 2002), which is mainly dependent on glucosyltransferase (GtfS) and glucan-binding protein (GbpS). Second, *S. mutans* metabolizes dietary sugars through lactate dehydrogenase (LDH) to produce lactic acid-based low pKa acid (Zeng and Burne, 2012). Under the restriction of the extracellular matrix, a local low-acid environment is formed and the function of the saliva buffer is impeded. When the pH of dental plaque falls below the critical threshold (pH = 5.5), it may lead to enamel dissolution and dental caries formation (Xiao et al., 2012). Finally, *S. mutans* has adapted to environmental changes and adverse factors within the biofilm. Specifically, to thrive in a low pH environment, *S. mutans* can activate an acid tolerance reaction (ATR) to protect itself from acid challenges (Baker et al., 2016).

Currently, the cariogenic signaling pathways of *S. mutans* include two-component signal transduction systems (TCSTS) (Matsumoto-Nakano, 2018), quorum sensing systems (QS) (Zu et al., 2019) and second messenger molecules (He et al., 2020). The VicRK system is a key TCSTS system that influences the adhesion, biofilm formation, EPS formation, acid production and acid resistance of *S. mutans* (Lei et al., 2019). The ComCDE system (QS system) primarily regulates the interspecies density sensing signal (Solano et al., 2014), plays an important role in biofilm formation, and is also involved in bacteriocin synthesis, and stress response (Shanmugam et al., 2020).

In recent years, non-cariogenic sucrose substitutes have garnered increased attention as alternative or auxiliary anti-caries compounds due to their availability, low toxicity and outstanding antibacterial and anti-biofilm capabilities (Durso et al., 2014). Stevioside is a diterpenoid compound, which is the main component of stevia extract (Goyal and Goyal, 2009). Its sweetness is approximately 300 times that of sucrose, but it possesses advantageous features such as low caloric content, resistance to high temperatures, and remarkable stability (Arumugam et al., 2020). Many researches indicate that it exhibits a range of beneficial properties including antibacterial, antioxidant, anti-cancer, lipid-lowering, antihypertensive, hypoglycemic, anti-tumor and diuretic effects, contributing to its medicinal value (Giacaman et al., 2013; Ramos-Tovar et al., 2018; Samuel et al., 2018). Previous studies on the anti-caries effect of stevioside mostly focused on planktonic bacteria (AlKanderi et al., 2023; Brambilla et al., 2013). Guo constructed a dual-species biofilm of *Streptococcus mutans* and *Candida albicans* (Guo et al., 2023). It was found that stevioside could inhibit the growth of bacteria in the mixed model. However, the conclusions about the interaction between *C. albicans* and *S. mutans* in oral biofilm caries are inconsistent. A study has shown that the synergy between *C. albicans* and *S. mutans* promotes biofilm maturation and maintains a lower acidic environment (Kim et al., 2021). Interestingly, Eidt observed that compared with dual-species biofilm (*S. mutans* and *C. albicans*), *S. mutans* single-species biofilm had lower pH and more serious enamel demineralization (Eidt et al., 2019). To explore the effect of stevioside on *S. mutans*, the study of a single-species model is essential. Thus, the anti-caries mechanism based on *S. mutans* biofilm and cariogenic virulence factors needs to be fully and deeply verified.

This study evaluated the effects of stevioside on biofilm formation, bacterial viability and virulence factors (EPS synthesis, acid production and acid resistance) of *S. mutans*. The study further compared the expression levels of genes associated with caries-related virulence factors, including *gtfB*, *gtfC*, *gtfD*, *ftf*, *ldh*, *atpF*, *comD*, *vicR*, *spaP*, and *gbpB* across different experimental groups. The findings presented in this paper elucidate the inhibitory effects of stevioside on the biofilm formation of *S. mutans* and explore the underlying mechanisms involved. Additionally, the study promotes subsequent research and application of stevioside as a natural non-cariogenic sucrose substitute.

## 2 Materials and methods

### 2.1 Bacterial strain and growth conditions

The standard strain of *S. mutans* (ATCC 25175) was obtained from Mingzhou Biotechnology Co., Ltd. (Ningbo, China). *S. mutans* was routinely inoculated in brain heart infusion (BHI) broth (Mingzhou Biotech Co., Ningbo, China) and incubated at 37 °C in an anaerobic environment (90%N_2_, 5%CO_2_, and5%H_2_). The standard strain was inoculated into BHI culture medium and anaerobically reactivated at 37 °C for 48 h. Following Gram staining and microscopic analysis, a pure culture was confirmed, and it was subsequently cultured on a solid plate for 24 h to isolate a single colony. Single colonies were picked and inoculated in BHI liquid medium overnight. After centrifugation, the precipitate was suspended and diluted with PBS to a concentration of 1 × 10^7^ CFU/mL, then stored at 4 °C for later use.

### 2.2 Experimental culture medium preparation

*Streptococcus mutans* biofilms were incubated in different solutions: BHI (as a control), BHI + 5% sucrose, BHI + 5% xylitol, and BHI + 5% stevioside. The concentrations of sucrose, xylitol and stevioside (5% wt/vol) were selected based on dose–response experiments (Cai et al., 2018).

### 2.3 Biofilm formation assay

The biofilm can be semi-quantitatively detected by crystal violet staining (Christensen et al., 1985). Standard bacterial suspension of 200 μL and 2 mL of BHI medium supplemented with different sugars were added to a 24-well plate, incubated at 37 °C for static incubation and changed every 24 h. The biomasses were assessed using crystal violet staining at 12, 24, 48, 72, and 96 h after incubation. First, the culture medium was discarded, and the biofilm at the bottom of the plate wells was washed slowly with sterile PBS buffer. Subsequently, the biofilm was fixed with formaldehyde solution. Next, the biofilm at the bottom of each hole was stained with 200 μL 0.1% crystal violet staining solution for 15 min and then washed twice with PBS after removing the solution. After completely drying, we added 500 μL 33% glacial acetic acid solution and treated it in a 37 °C incubator for 20 min to dissolve crystal violet. Finally, 200 μL solution per hole of a 24-well plate was transferred to the new 96-well plate, and the microplate reader read the absorbance value at 575 nm to quantify the biofilm.

### 2.4 Acid production from biofilms

The standard bacterial suspension was inoculated into BHI medium supplemented with sucrose, xylitol, and stevioside at 1:10 (v:v). The mixture was anaerobically cultured at 37 °C. The supernatant of each group was collected and centrifuged at 6,000 g for 10 min to remove bacterial precipitation. The pH of the supernatant after centrifugation was measured by a pH meter (Mettler-Toledo, Columbus, United States) at 0, 12, 24, 48, 72, and 96 h after incubation.

### 2.5 Lactic acid assay

A 200 μL aliquot of standard bacterial suspension was mixed with 2 mL of BHI broth supplemented with different sugars and incubated anaerobically at 37 °C for 96 h to allow biofilm formation. After glycolysis, the supernatant from each group was collected, centrifuged at 6,000 × g for 10 min to remove bacterial pellets, and stored at 4 °C for further analysis. Lactic acid content in the supernatant was quantified using a commercial detection kit (Jiancheng, Nanjing).

### 2.6 CFU counting for quantification of biofilm biomass

The continuous dilution technique is helpful for the simultaneous evaluation of microbial populations and counts (Harris and Sommers, 1968). The standard bacterial suspension of 200 μL was combined with 2 mL of BHI containing different sugars, and subsequently incubated anaerobically at 37 °C for 96 h to facilitate biofilm formation. Following incubation, the culture medium was removed, and the biofilm at the bottom of the well was gently rinsed with sterile PBS to remove the residual culture medium and unattached bacteria on the biofilm. The biofilm was then scraped off, collected, and resuspended in PBS. The biofilm suspension was vortexed thoroughly to achieve complete dispersion into individual cells, facilitating accurate counting on the coating plate. An equal volume of the biofilm suspension from each experimental group was diluted from 10^4^-fold to 10^6^-fold with PBS buffer and evenly coated onto BHI agar plates with sterile L rods. The colonies were counted after anaerobic incubation at 37 °C for 48 h.

### 2.7 Exopolysaccharide measurements

The anthrone-sulfuric acid method was used to determine the amount of IEPS and SEPS in biofilms (Koo et al., 2010a,b). Briefly, bacterial suspension and BHI with or without sugar were mixed to a 24-well plate and cultured for 4 days at 37 °C. We removed the culture medium and then gently washed the biofilm with PBS. Biofilms were collected by scraping with same voiume of PBS, and were centrifuged (6,000 g, 4 °C, 10 min) to separate IEPS and SEPS. SEPS was located in the supernatant, and IEPS was in the precipitate. The supernatant was transferred to a new centrifuge tube. The precipitate was resuspended in PBS and centrifuged. The centrifugation method was the same as before, and the supernatant was discarded. The precipitate was resuspended in 0.4 M NaOH and incubated at 37 °C for 2 h. After centrifugation (6,000 g, 4 °C, 10 min), the supernatant was collected to obtain IEPS. Then, 250 μL of SEPS or IEPS and 750 μL of anthrone-sulfuric acid solution were added to the new centrifuge tube, and the mixture was heated at 95 °C for 10 min in a dry heater and cooled at room temperature. We transferred 200 μL of the mixture to a 96-well plate and detected the absorbance value of the sample at 625 nm using a microplate reader. Finally, the corresponding polysaccharide content was calculated according to the glucose standard curve.

### 2.8 Scanning electron microscopy observation of biofilm

The morphological and physiological characteristics of *S. mutans* biofilm were observed by scanning electron microscopy (SEM). After UV disinfection for 2 h, round-shaped glass slides were placed on a 24-well plate. The standard bacteria suspension and medium solution were added to the plate at 1:10 (v/v) for 4 days. Then, the medium was discarded and the biofilm-loaded slides were washed 3 times with sterile PBS. The slide was transferred to a new 24-well plate and completely covered with 2.5% (v/v) glutaraldehyde. The biofilm was fixed at 4 °C for 10 h in the dark, then washed gently with PBS. Next, the biofilm was dehydrated in a series of ethanol concentrations (30, 40, 50, 60, 70, 80, 90, 100%) every 15 min. Finally, the glass is dried. After spraying gold, the samples were examined by a scanning electron microscope. Three points were randomly selected in each sample and observed at three magnifications (×2,000, ×5,000).

### 2.9 Live/dead bacteria staining

SYTO-9 and PI nucleic acid dyes (Invitrogen, United States) performed double fluorescence staining on the biofilm. The former made the living bacteria stained with green fluorescence, and the latter penetrated the damaged cell membrane to make the dead bacteria present red fluorescence. The method of biofilm formation has been described by SEM. The glass slides containing the biofilm were placed in a sterile disposable dish and then washed with PBS. Then, the dyeing working solution was prepared according to the instructions, and it was gently dripped onto the surface of the sample, and dyed at 37 °C for 15 min in the dark. We used a laser confocal laser scanning microscopy (Olympus FV1200, Japan) to capture biofilm images. Three points were randomly selected for each sample, and Image J software was used to quantitatively evaluate the fluorescence intensity and thickness of the biofilm image.

### 2.10 RT–qPCR analysis of cariogenic gene expression in biofilms

Standard bacteria suspension was grown in BHI supplemented with different sugars and incubated for 4 days anaerobically at 37 °C. The adherent biofilm was collected by the same CFU experimental method. The bacterial precipitate was resuspended in 200 μL (100 mg/mL, Biofroxx) of lysozyme, incubated in a 37 °C water bath for 3 h, and vortexed every half an hour. The total RNA was extracted according to the instructions (NCM Biotech, China), and the purity (A260/A280) and concentration of total RNA were determined by NanoDrop 2000 spectrophotometer (Thermo Scientific, United States). cDNA was synthesized by a HiScript IV RT SuperMix for qPCR (Vazyme, Nanjing, China). Real-time quantitative PCR amplification was performed using Taq Pro Universal SYBR qPCR Master Mix (Vazyme, Nanjing, China).

The primer design involved in this experiment is based on the previous experiment of our research group (Guan et al., 2020), and the primer sequence is shown in Table 1. We detected 10 target genes related to *S. mutans* biofilm glucan production, fructose production, acid production, acid resistance, two-component signal and quorum sensing. We analyzed them using the QuantStudio 5 real-time fluorescence quantitative PCR system (Thermo Scientific, United States) and software. The gene expression was normalized with reference gene 16S rRNA using the 2^−ΔΔCT^ method.

**Table 1:** Nucleotide sequences of primers used in this study.

Gene	Description	Primer sequence (5′-3′)
*16SrRNA*	Normalizing internal standard	F: CCTACGGGAGGCAGCAGTAG R: CAACAGAGCTTTACGATCCGAAA
*spaP*	Surface-associated protein P1	F: TCCGTGCCGATAATCCAAGA R: CGCCTGTTTGTCCCATTTGT
*gbpB*	Glucan binding production	F: AGCAGCGGCAGGATATAGAG R: ACCAACCACGGTAGTTACCAATA
*gtfB*	Water insoluble glucan production	F: GGTCACTGGTGCTCAATCAAT R: AAGCGTAAGTTCCATCTTCATTCT
*gtfC*	Water insoluble and soluble production	F: TCAGACAACACCTTCCTTCCTA R: GAGCACCAGTGACCATATAACC
*gtfD*	Water soluble glucan production	F: GCCTTTTTACGCTTGTTTGT R: CCATATTCATATTCTCCGCC
*ftf*	Fructosyltransferase	F: CGAACGGCGACTTACTCTTAT R: TTACCTGCGACTTCATTACGATT
*ldh*	Lactate dehydrogenase	F: TTGCTCGTATCACTAAGGCTATTC R: GGGCTGACCGATAAAGACTTC
*atpF*	H^+^-translocating F-ATPase	F: TTGATAACGCTAAGGAAACTGGTA R: AACGCTTGATAGGGCTTCTG
*comD*	Competence-stimulating peptide	F: ATGGTCTGCTGCCTGTTG R: CGATCATATAGGTGGTTA
*vicR*	Response regulator	F: GCATCACTTAGCGACACACA R: CAGACGACGAACAGTAACATCAA

### 2.11 Data analysis

Results were expressed as mean with SD. Each experiment was replicated independently three times. We used SPSS 25.0 for statistical analysis and GraphPad Prism 9 for graphing. One-way ANOVA and Tukey test were used to determine the statistical significance of each group of differences. Different letters indicate significant differences among different treatment groups. The specific statistical significance is marked with asterisks.

## 3 Results

### 3.1 Biofilm formation

**Figure 1:**
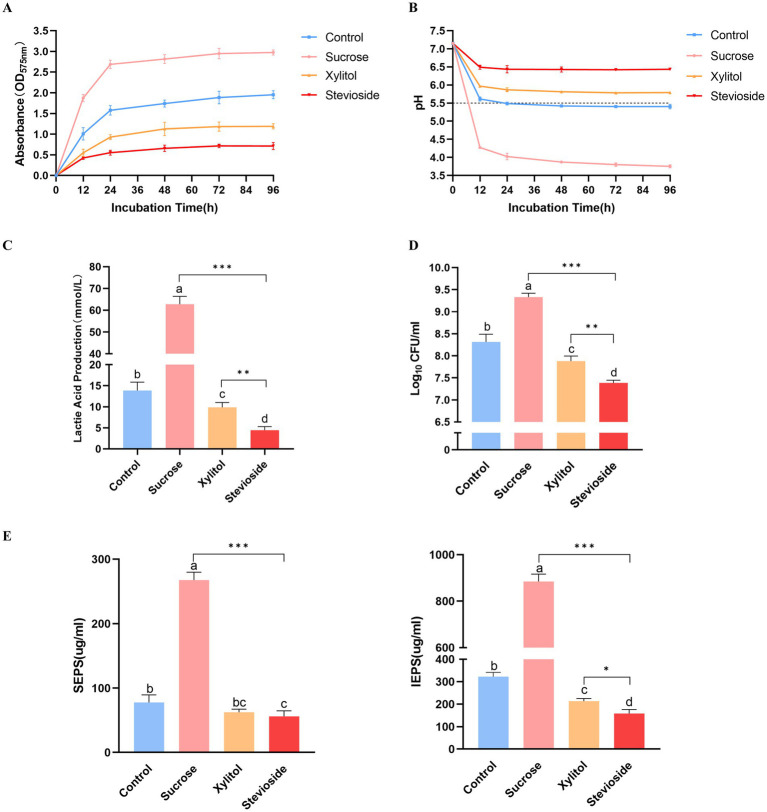
**(A)** Growth curve of *Streptococcus mutans* biofilm added with extra sugar. **(B)** Different sugar-containing culture medium decreased the glycolysis pH of *Streptococcus mutans* biofilm. The horizontal dotted line represents the critical pH value of demineralization (pH 5.5). **(C)** Lactic acid production by biofilm bacteria cultured for 96 h. **(D)** The bacterial activity of *Streptococcus mutans* biofilm was measured by CFU count. **(E)** The yield of SEPS and IEPS was determined by anthrone-sulfuric acid method. The data were expressed as mean ± standard deviation. Different letters represent significant differences among treatments. Statistical significance is marked with asterisk: *p < 0.05, **p < 0.01, ***p < 0.001.

Crystal violet staining showed that the amount of *S. mutans* biofilm formation was shown in Figure 1A. The amount of biofilm in the sucrose group was significantly higher than that in the xylitol and stevioside groups at each time point (*p* < 0.05). At the early stage of biofilm formation (12 h), the sucrose group had formed more biofilms and basically stabilized at 96 h. The amount of biofilm formed in the xylitol group from 12 h was significantly less than that in the control group (*p* < 0.05). There was no significant difference in the amount of biofilm between the xylitol group and the stevioside group at 12 h (*p* > 0.05). However, the biofilm formation in the stevioside group was less than that in the xylitol and sucrose groups at 24 h (*p* < 0.05), and the inhibitory effect was stable until 96 h.

### 3.2 Acid production of biofilm

The acid production capacity in different growth environments was evaluated by measuring pH (Figure 1B). The initial pH value of the culture medium in this study was 7.15 ± 0.02. In the presence of sucrose, the pH of the medium decreased sharply after 12 h, and the lowest pH was maintained until 96 h compared with other groups (*p* < 0.05). In addition, the pH of the sucrose group decreased gradually with time from 12 h to 48 h (*p* < 0.05), and there was no significant difference between the pH of 72 h and 96 h and 48 h (*p* > 0.05). The pH of the xylitol group was higher than that of the control group at each time point (*p* < 0.05). At the same time point, the pH value of the stevioside group remained at the highest level throughout the experiment compared with the other three groups (*p* < 0.05). However, there was no significant difference in the pH value of the stevioside group at different time points (*p* > 0.05).

### 3.3 Lactic acid assay

The lactic acid production levels were measured across different experimental groups (Figure 1C). The sucrose group exhibited the highest lactic acid production (*p* < 0.001). The xylitol group showed lower lactic acid levels than the control group (*p* < 0.05), while the stevioside group demonstrated even further reduction compared to the xylitol group (*p* < 0.01).

### 3.4 Biofilm bacteria viability

The bacterial viability in the biofilm was quantitatively evaluated by CFU count (Figure 1D). In the presence of sucrose, the bacterial viability in the biofilm was the highest, significantly higher than that in the control, xylitol, and stevioside groups (*p* < 0.05). The bacterial viability of biofilm in the xylitol group was lower than that in the control group (*p* < 0.05), but higher than that in the stevioside group (*p* < 0.01). In the stevioside group, the effect of inhibiting biofilm bacterial viability was significantly higher than that of other groups (*p* < 0.01).

### 3.5 Evaluation of extracellular polysaccharide production

The exopolysaccharides in biofilms were quantitatively determined by the anthrone-sulfuric acid method (Figure 1E). The SEPS and IEPS of the sucrose group were significantly higher than those groups (*p* < 0.001). This observation also corresponded to the SEM images of the sucrose group in which the bacteria were wrapped in the three-dimensional framework formed by exopolysaccharides. There was no significant difference in the content of SEPS between the xylitol group and the control group (*p* > 0.05), but the content of IEPS was lower in the xylitol group than in the control group (*p* < 0.05). In the presence of stevioside, the content of SEPS was not significantly different from that of the xylitol group (*p* > 0.05), but IEPS content was lower than that of the xylitol group (*p* < 0.05).

### 3.6 SEM observation

**Figure 2:**
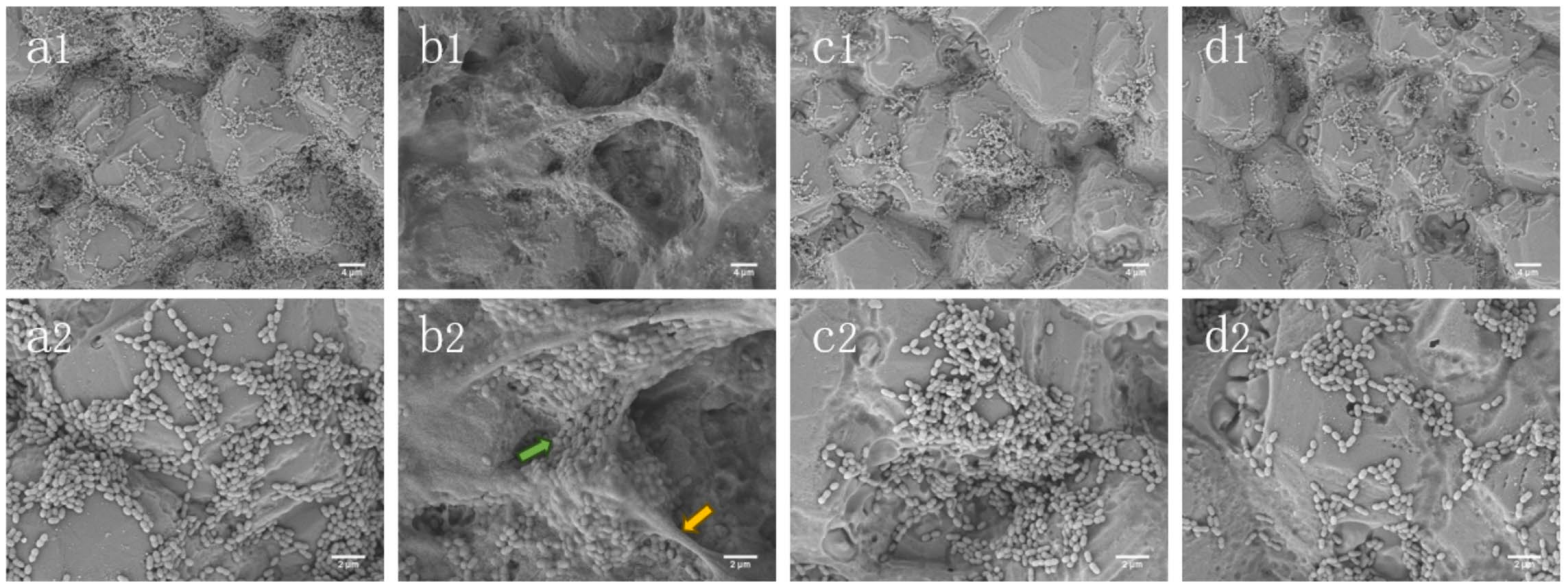
SEM images of *Streptococcus mutans* biofilms. **(a1, a2)** Control; **(b1, b2)** Sucrose; **(c1, c2)** Xylitol; **(d1, d2)** Stevioside. The images were magnified by 2,000 and 5,000 times, respectively. The yellow arrow represents EPS, while the green arrow represents bacteria.

The biofilm formed by *S. mutans* biofilm on glass slides was observed by SEM (Figure 2). In the presence of sucrose, the number of *S. mutans* increased significantly, and sucrose could be metabolized to produce a large number of extracellular substances. The bacterial cells were encapsulated in a three-dimensional structure formed by many extracellular substances. The control group had no obvious three-dimensional structure, fewer bacteria were distributed in a flat shape, and no obvious extracellular matrix was observed. The number of *S. mutans* in the xylitol group was lower than that in the sucrose group and the control group, and the extracellular matrix on the surface of the bacteria was also rare. Compared with the other three groups, the stevioside group was significantly reduced, not distributed in clusters, and observed in short-chain arrangement under high magnification.

### 3.7 Live/dead bacteria staining

**Figure 3:**
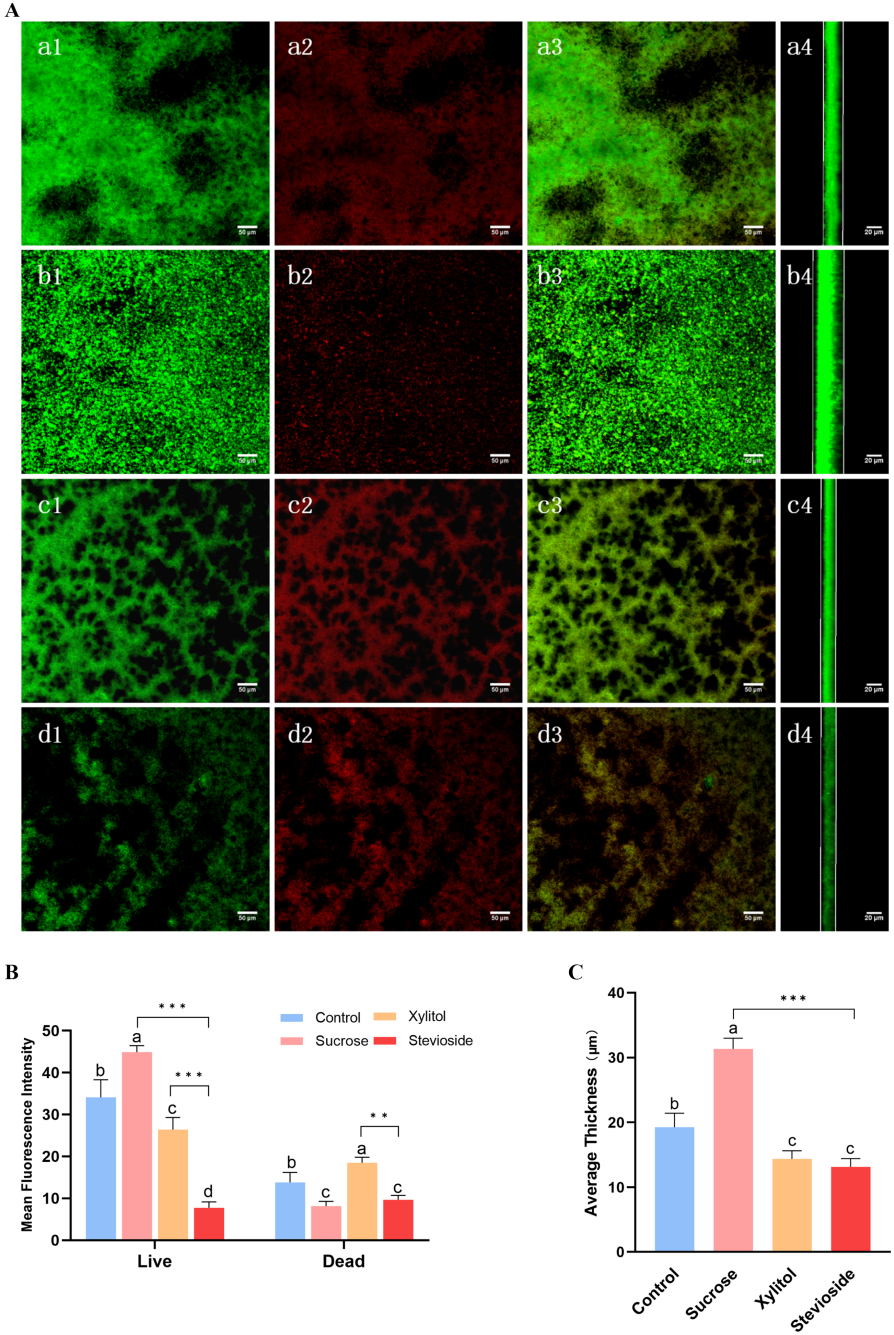
Stevioside has anti-biofilm activity against *Streptococcus mutans* biofilm. After staining with LIVE/DEAD fluorescent dye (SYTO9/PI), the biofilm was observed by confocal microscopy. **(A)** Green: living cells. Red: dead cells. Mix: live + dead cells. The side represents the thickness of the biofilm. **(a1–a4)** Control; **(b1–b4)** Sucrose; **(c1–c4)** Xylitol; **(d1–d4)** Stevioside. Image J was used to collect and analyze the fluorescence images. The fluorescence intensity of living cells and dead cells **(B)** and average thickness **(C)** of the treated biofilm were compared. The data were expressed as mean ± standard deviation. Different letters represent significant differences among treatments. Statistical significance is marked with asterisk: *p < 0.05, **p < 0.01, ***p < 0.001.

As shown in Figure 3, *S. mutans* biofilm was stained with live/dead bacteria, SYTO-9 dye stained the live bacteria green, and PI stained the bacteria red. In Figure 3A, the biofilm of the sucrose group was mostly green and thick, covering nearly the entire field of view. In contrast, the control group exhibited a reduction in both the green area and thickness of the biofilm. When compared to the control group, the xylitol group showed a decrease in biofilm area, with a higher number of dead bacteria than live ones, resulting in a slightly orange biofilm mixture. The stevioside group demonstrated a significant reduction in biofilm, and the bacterial mixture map displayed a distinct orange color.

As shown in Figure 3B, the biomass of biofilm in the sucrose group (green fluorescence) was the highest (*p* < 0.05), and that in the control group, xylitol group and stevioside group decreased in turn (*p* < 0.05), which was consistent with the results of biofilm formation. In Figure 3B, the fluorescence intensity of dead bacteria in the sucrose group was lower than that in both the xylitol and control groups (*p* < 0.05). The proportion of dead bacteria in the stevioside group was lower than that in the xylitol group (*p* < 0.05). It is hypothesized that this phenomenon may be attributed to a reduced biofilm area in the stevioside group, leading to a diminished overall fluorescence intensity for dead bacteria. In Figure 3C, the biofilm thickness of the sucrose group was the thickest (*p* < 0.05), measuring approximately 31.35 ± 1.63 μm. Compared with the control group, the biofilm thickness of the xylitol and stevioside groups decreased (*p* < 0.05). The thickness of the stevioside group was the least, but there was no significant difference between the stevioside group and the xylitol group (*p* > 0.05).

### 3.8 Gene expression of cariogenic virulence

**Figure 4:**
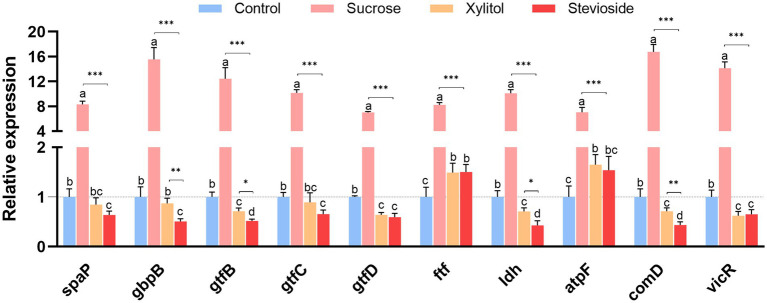
RT-qPCR analysis showed that the medium supplemented with sucrose, xylitol and stevioside changed the expression of genes related to the cariogenic virulence factors of *Streptococcus mutans* biofilm. The data were expressed as mean ± standard deviation. Different letters represent significant differences among treatments. Statistical significance is marked with asterisk: *p < 0.05, **p < 0.01, ***p < 0.001.

As shown in Figure 4, RT-qPCR results showed the relative expression levels of cariogenic virulence-related genes in *S. mutans* biofilms. The expression of the *spaP* gene was related to the non-specific adhesion of bacteria. In the presence of sucrose, the expression of this gene was significantly up-regulated (*p* < 0.001). There was no notable difference in *spaP* gene expression between the stevioside and xylitol groups (*p* > 0.05). Genes related to polysaccharide synthesis and glucan binding protein (*gtfB*, *gtfC*, *gtfD*, *ftf*, *gbpB*) were also significantly up-regulated in the sucrose group (*p* < 0.05). The *gtfB* gene in the stevioside group was down-regulated compared with the xylitol group (*p* < 0.05). There were no significant differences in the expression of *gtfC*, *gtfD* and *ftf* genes between the stevioside and the xylitol groups (*p* > 0.05), but the *gbpB* gene expression was lower than that of the xylitol group (*p* < 0.01). The expression of the *ldh* and *atpF* genes are related to acid production and resistance of *S. mutans*. The *ldh* gene in the stevioside group was down-regulated compared to the sucrose group (*p* < 0.001) and xylitol group (*p* < 0.05). The *atpF* gene expression was lower than that of the sucrose group (*p* < 0.001), but there was no significant difference with the xylitol group (*p* > 0.05). The transcription levels of the two signaling pathway-related genes *comD* and *vicR* were highest in the sucrose group (*p* < 0.001). The expression of the *comD* gene in the stevioside group was lower than that in the xylitol group (*p* < 0.01), while the expression of *vicR* did not significantly differ from that in the xylitol group (*p* > 0.05).

## 4 Discussion

Dental caries constitutes a prevalent oral microbial and glucose-mediated biofilm-dependent oral disease (Pitts et al., 2017). Among the many sugar substances, sucrose is the most important cariogenic sugar, playing a crucial role in the metabolic processes of *S. mutans* (Sheiham and James, 2015). Therefore, substituting sucrose with anti-cariogenic sweeteners holds great promise for preventing dental caries. Xylitol, while widely utilized in various food products (Nayak et al., 2014), presents certain disadvantages, including elevated production expenses, the possibility of gastrointestinal disturbances with extended consumption, and the development of xylitol-resistant mutants (Meurman et al., 2005). Consequently, the exploration of innovative natural substitutes for sucrose is essential. Presently, while investigations into the impact of stevioside on cariogenic bacteria have hinted at its certain anti-cariogenic potential (Ajagannanavar et al., 2014; Escobar et al., 2020), the constraints inherent in experimental designs necessitate more comprehensive scientific and clinical-related studies to fully unlock the capabilities of stevioside within anti-cariogenic strategies.

To explore more about the inhibitory mechanism of stevioside on the biofilm formation of *S. mutans*, in this study, we observed that stevioside could inhibit the formation of total biomass of *S. mutans* biofilm (Figure 1A). We speculate that this inhibitory phenomenon be attributed to either a diminution in the bacterial cell count or an interference with the bacterial adhesion process. Subsequently, we found that stevioside could inhibit bacterial growth and reduce the number of viable cells (Figure 1D). This finding was further corroborated by CLSM, which likewise detected a discernible decline in the viable cell population (Figure 3B). Moreover, the genes regulating GbpB (glucan-binding protein) (Matsumura et al., 2003) and SpaP (surface adhesion protein) (Manzer et al., 2020) in this experiment were much higher in the sucrose group than in the stevioside group (Figure 4), indicating that the inhibition of adhesion gene expression by stevioside may help reduce bacterial surface adhesion and affect biofilm formation.

Extracellular polysaccharides account for 40% of the dry weight of biofilm, which are usually synthesized by microbial glucosyltransferase (Koo et al., 2010a,b). Therefore, the decrease of biofilm biomass is directly related to the decrease of polysaccharide levels in the whole biofilm matrix. In our study, xylitol and stevioside significantly reduced the production of IEPS and SEPS compared with sucrose and control groups, indicating that they can specifically target the synthesis of EPS, thereby affecting the integrity of biofilms (Figure 1E). Complementary to these findings, SEM images provided further insights into the structural differences among the groups (Figure 2). The stevioside group exhibited a much sparser distribution of bacteria, which were predominantly in short-chain and dispersed formations, and no conspicuous extracellular polymers were detected. Gene expression analysis further confirmed this point. Compared with sucrose, stevioside inhibited the expression of *gtfB*, *gtfC* and *gtfD* (EPS-related) (Figure 4); compared with xylitol, stevioside did not inhibit *gtfD* responsible for SEPS synthesis (Figure 4). In the ALKanderi study (AlKanderi et al., 2023), different concentrations of stevia extracts could inhibit IEPS compared with the control group, but the synthesis of SEPS was higher than that of the control group. Guo found that stevioside reduced sucrose production by EPS staining, which was not significantly different from the TSB and xylitol groups (Guo et al., 2023). In this study, the production of SEPS and IEPS was quantitatively studied by establishing a glucose standard curve and combining with the anthrone sulfuric acid method.

*Streptococcus mutans* metabolizes carbohydrates, activates lactate dehydrogenase (high carbohydrate utilization), produces organic acids, and reduces the pH of dental plaque (Takahashi, 2015). The pH in the sucrose group was observed to drop to approximately 4, while the pH of the stevioside group consistently remained above the critical threshold for demineralization (Figure 1B). Moreover, the expression of the *ldh* gene was detected at the molecular mechanism level, and it was found that stevioside downregulated the acid-producing gene (Figure 4). We speculate that the decrease in gene expression might lead to a reduction in the content of the regulated LDH. However, more precise research is required to explore the activity of lactate dehydrogenase. The gene *atpF*, encoding membrane-bound F-ATPase, plays a crucial role in the molecular mechanism of acid resistance response (ATR) (Welin-Neilands and Svensäter, 2007). In this study, the expression of the *atpF* gene in the stevioside group was lower than that in sucrose (Figure 4), indicating that stevioside may inhibit associated genes and heighten susceptibility to acid stress.

The TCSTS functions as a molecular switch, enabling bacteria to detect external environmental changes and modulate internal gene expression (Kawada-Matsuo and Komatsuzawa, 2017). The gene *vicR* encodes an intracellular response regulatory protein capable of modulating the expression of *gbpB*, *gtfB*, *gtfC*, and *gtfD* upon phosphorylation (Stipp et al., 2013). The gene *comD* encodes a histidine protein kinase (Kaur et al., 2015). It is part of the quorum sensing (QS) cascade of *S. mutans* and plays an important role in biofilm formation and enhancing the ecological advantages of *S. mutans*. SEM (Figure 2) and CLSM (Figure 3A) images showed that the biofilm area of the stevioside group was notably smaller than that of the sucrose group, and the EPS production of the stevioside group was decreased by the anthrone-sulfuric acid method (Figure 1E). Based on these findings, we speculated that stevioside may affect biofilm formation and extracellular polysaccharide production by down-regulating *comD* and *vicR* gene expression (Figure 4).

However, despite the rich microbial species in the artificial biofilm model, there are still limitations compared with natural oral biofilms (Marsh, 2010). The balance of the oral microecology depends on the synergy between commensal bacteria (such as *Streptococcus sanguinis*, *Streptococcus gordonii*, etc.) and the host. These bacteria maintain a healthy oral microenvironment by competitively inhibiting the adhesion of pathogenic bacteria and secreting antimicrobial substances. It can be inferred from this study that stevioside may help commensal bacteria exert their functions: after inhibiting the formation of *Streptococcus mutans* biofilm, it can reduce the competition of pathogenic bacteria for nutrients and adhesion sites, provide a more favorable living environment for commensal bacteria, and indirectly enhance the stabilizing effect of commensal bacteria on the oral microenvironment. However, the specific effects of stevioside on different commensal bacteria still need to be further clarified through more *in vitro* multispecies co-culture experiments and *in vivo* studies. This will make stevioside expected to be used as an effective medical excipient, which can be applied to various oral care products and the formulation of caries prevention and treatment drugs, to improve the palatability of drugs, and assist in the regulation of oral microecology, and fundamentally prevent the occurrence of dental caries.

## 5 Conclusion

In summary, stevia extract, stevioside, effectively inhibited biofilm formation and growth, acid production, bacterial activity and extracellular polysaccharide production, and destroyed the structural integrity of biofilm. Furthermore, stevioside suppressed the expression of cariogenic virulence factor genes (*gtfB*, *gtfC*, *gtfD*, *ftf*, *spaP*, *gbpB*, *ldh*, *atpF*, *comD*, and *vicR*) related to adhesion, EPS synthesis, acid production and acid resistance, and biofilm formation and maturation. Based on biofilm and cariogenic virulence factors, the results of this experiment suggest that stevioside may inhibit the cariogenic properties of *S. mutans*, indicating that stevioside has the potential to become a new generation of natural non-cariogenic sucrose substitutes.

## Data Availability

The raw data supporting the conclusions of this article will be made available by the authors, without undue reservation.
