# Supplementary figures and images for: Inhibition of Streptococcus mutans biofilm formation and virulence by natural extract Stevioside
Source: Front Microbiol. 2025 Oct 2;16:1675322. doi: 10.3389/fmicb.2025.1675322 (PMC12528021; doi:10.3389/fmicb.2025.1675322)

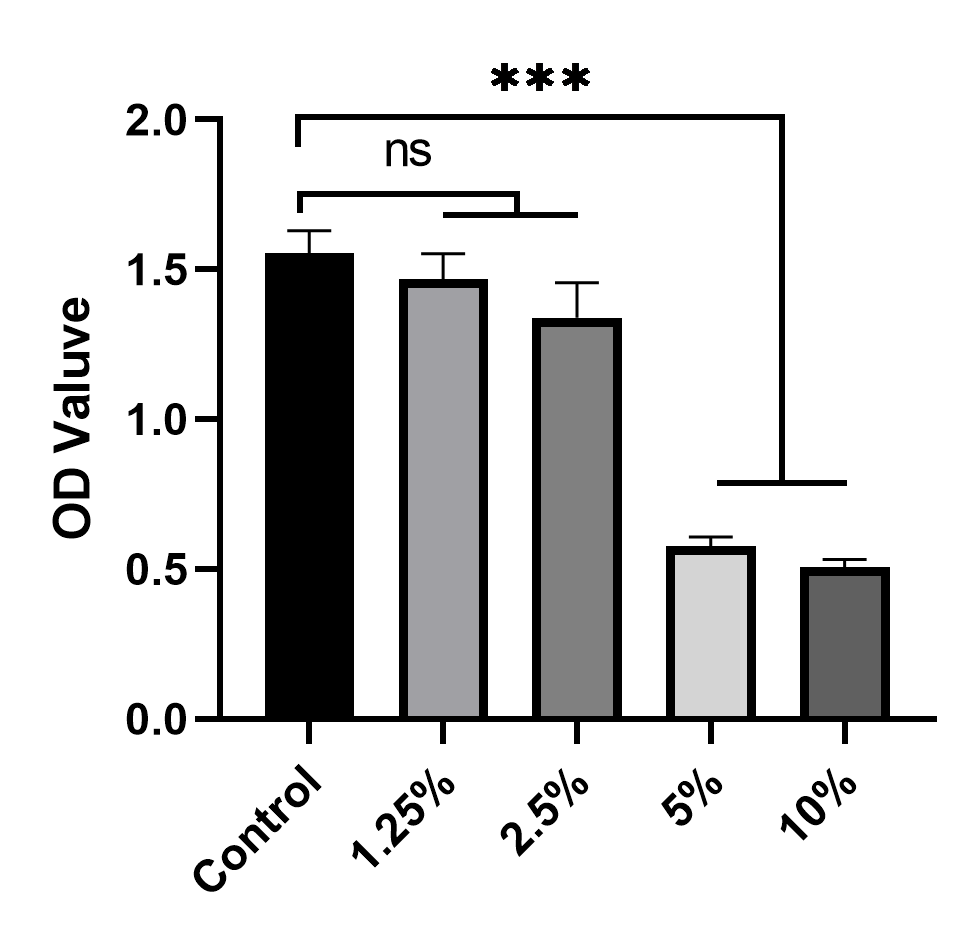

Supplement: Supplementary file 1 [file Image_1.TIF]
